# Tetra­butyl­ammonium hydrogen phenyl­arsonate–phenyl­arsonic acid (1/1)

**DOI:** 10.1107/S1600536812035362

**Published:** 2012-08-25

**Authors:** Lukas Reck, Wolfgang Schmitt

**Affiliations:** a School of Chemistry, Trinity College, Dublin 2, Ireland

## Abstract

The structure of the title salt adduct, (C_4_H_9_)_4_N^+^·C_6_H_5_AsO_3_H^−^·C_6_H_5_AsO_3_H_2_, features chains along the *a* axis comprising alternating hydrogen phenyl­arsonate anions and phenyl­arsonic acid mol­ecules linked by O—H⋯O hydrogen bonds.

## Related literature

For similar structures containing bulky hydro­phobic cations and hydrogen-bonded chains of hydrogen(aryl­phospho­nate)/aryl­phospho­nic acid, see: Clarke *et al.* (2005 ▶); Latham *et al.* (2007 ▶, 2008 ▶). For hybrid organic–inorganic polyoxidometalate frameworks including aryl­arsonic acid ligands, see: Breen, Clérac *et al.* (2012 ▶); Breen, Zhang *et al.* (2012 ▶); Zhang & Schmitt (2011 ▶); Onet *et al.* (2011 ▶); Breen & Schmitt (2008 ▶).
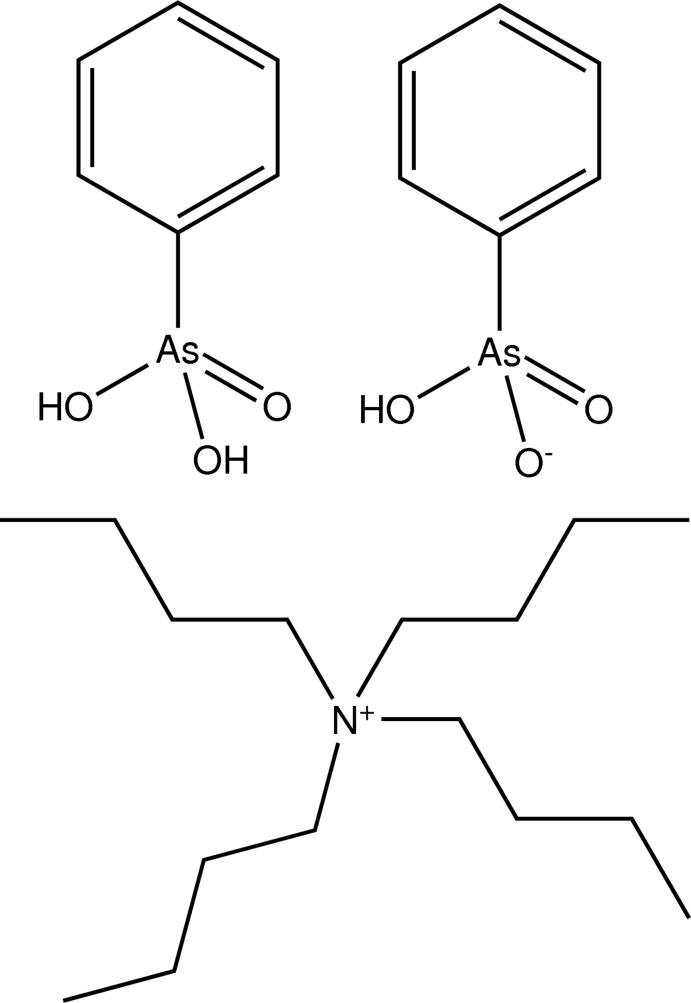


## Experimental

### 

#### Crystal data

C_16_H_36_N^+^·C_6_H_6_AsO_3_^−^·C_6_H_7_AsO_3_
*M* *_r_* = 645.52
Triclinic, 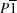

*a* = 9.035 (2) Å
*b* = 10.137 (3) Å
*c* = 18.789 (5) Å
α = 94.005 (5)°
β = 97.749 (4)°
γ = 114.289 (4)°
*V* = 1539.2 (7) Å^3^
*Z* = 2
Mo *K*α radiation
μ = 2.21 mm^−1^
*T* = 120 K
0.5 × 0.3 × 0.1 mm

#### Data collection

Bruker SMART APEX diffractometer
Absorption correction: multi-scan (*SADABS*; Bruker, 1997 ▶) *T*_min_ = 0.291, *T*_max_ = 0.809
15253 measured reflections
7513 independent reflections
6769 reflections with *I* > 2σ(*I*)
*R*_int_ = 0.023

#### Refinement

*R*[*F* ^2^ > 2σ(*F* ^2^)] = 0.026
*wR*(*F* ^2^) = 0.070
*S* = 1.03
7513 reflections
530 parameters
All H-atom parameters refined
Δρ_max_ = 0.61 e Å^−3^
Δρ_min_ = −0.71 e Å^−3^

### 

Data collection: *SMART* (Bruker, 1997 ▶); cell refinement: *SAINT* (Bruker, 1997 ▶); data reduction: *SAINT*; program(s) used to solve structure: *SHELXS97* (Sheldrick, 2008 ▶) and *OLEX2* (Dolomanov *et al.*, 2009 ▶); program(s) used to refine structure: *SHELXL97* (Sheldrick, 2008 ▶) and *OLEX2*; molecular graphics: *OLEX2*; software used to prepare material for publication: *SHELXL97* and *OLEX2*.

## Supplementary Material

Crystal structure: contains datablock(s) I, global. DOI: 10.1107/S1600536812035362/tk5138sup1.cif

Structure factors: contains datablock(s) I. DOI: 10.1107/S1600536812035362/tk5138Isup2.hkl

Supplementary material file. DOI: 10.1107/S1600536812035362/tk5138Isup3.cdx

Additional supplementary materials:  crystallographic information; 3D view; checkCIF report

## Figures and Tables

**Table 1:** Selected bond lengths (Å)

As1—O13	1.6625 (10)
As1—O12	1.6723 (11)
As1—O11	1.7279 (11)
As1—C11	1.9001 (16)
As2—O23	1.6432 (11)
As2—O22	1.7013 (11)
As2—O21	1.7030 (12)
As2—C21	1.9153 (16)

**Table 2:** Hydrogen-bond geometry (Å, °)

*D*—H⋯*A*	*D*—H	H⋯*A*	*D*⋯*A*	*D*—H⋯*A*
O11—H11⋯O23	0.77 (3)	1.88 (3)	2.6375 (17)	166 (3)
O21—H21⋯O13^i^	0.75 (3)	1.78 (3)	2.5280 (17)	176 (3)
O22—H22⋯O12^i^	0.93 (4)	1.57 (4)	2.4936 (17)	176 (4)

Symmetry code: (i) 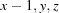.

**Fig. 1.**
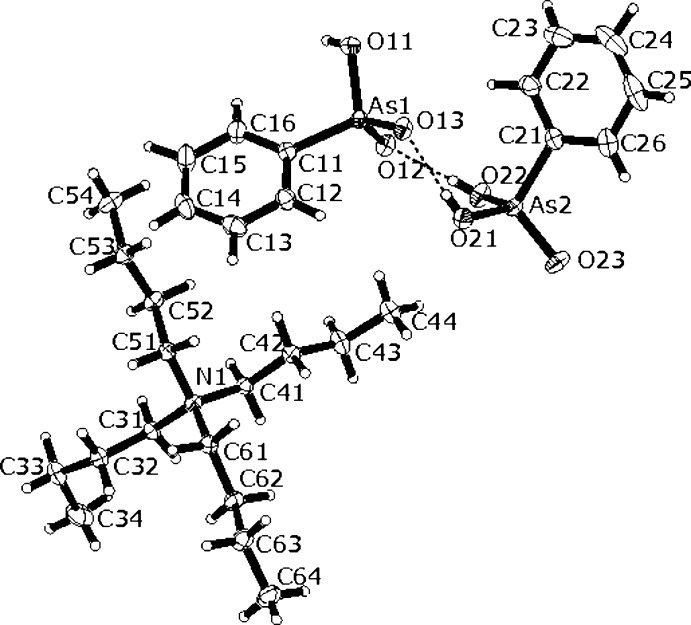
The asymmetric unit of the title compound, showing the numbering scheme and displacement ellipsoids on non-H atoms at the 50% probability level.

**Fig. 2.**
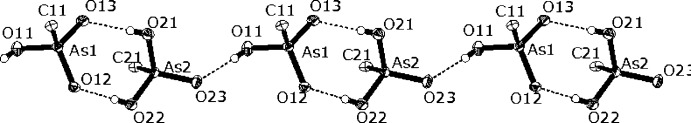
A hydrogen-bonded one-dimensional chain of alternating hydrogen phenylarsonate anions and phenylarsonic acid molecules. Phenyl rings (except for the carbon atom bound to As) and counterions have been omitted for clarity. Displacement ellipsoids on non-H atoms are drawn at the 50% probability level.

## supplementary crystallographic information

### Comment

In the course of our studies on hybrid organic-inorganic polyoxometalate frameworks, including arylarsonic acid ligands (Breen, Clérac *et al.*, 2012; Breen, Zhang *et al.*, 2012; Zhang & Schmitt, 2011; Onet *et al.*, 2011; Breen & Schmitt, 2008), we attempted to prepare tetrabutylammonium hydrogen phenylarsonate [(C_4_H_9_)_4_N]^+^[C_6_H_5_AsO_3_H]^-^ as a starting material for synthesis. Unexpectedly, mixing equimolar amounts of tetrabutylammonium hydroxide and phenylarsonic acid in aqueous solution and slow evaporation resulted in crystals with a 2:1 stoichiometry of tetrabutylammonium to phenylarsonic acid. We therefore undertook a closer structural examination of these crystals in order to find out if there is a structural reason for the apparent stability of this stoichiometry.

The structure consists of hydrogen-bonded chains of alternating hydrogen phenylarsonate anions and phenylarsonic acid molecules extending in the direction of the crystallographic *a* axis. These chains form two-dimensional sheets *via*π-π interactions that extend in the crystallographic (010) plane. The sheets alternate along the crystallographic *b* axis with layers consisting of tetrabutylammonium cations to form a lamellar structure. Analogous structures are known for salts with a 2:1 stoichiometry between a bulky organic monocation and phenylphosphonic acid (see Clarke *et al.*, 2005, Latham *et al.*, 2007 and Latham *et al.*, 2008), but to our knowledge, this is the first time it has been reported for phenylarsonic acid.

The hydrogen phenylarsonate anion contains one long and two short As–O bonds, while the phenylarsonic acid molecule contains one short and two long As–O bonds (see Table 1). This is consistent with the assigned proton positions on these molecules: the As–O distance is shorter when the oxygen atom is unprotonated, as the formal bond order of these bonds is higher than that of bonds to protonated oxygen atoms.

Each hydrogen mphenylarsonate anion acts as a hydrogen acceptor for two very short hydrogen bonds from one neighbouring phenylarsonic acid molecule and as a hydrogen bond donor for a longer hydrogen bond to the other neighbouring phenylarsonic acid molecule (see Table 2, Figure 2).

π-π interactions are weak, with the centroid to centroid distance being 3.8669 (17) Å between the phenyl ring on the hydrogen mphenylarsonate anion and its closest symmetry equivalent and 4.0264 (17) Å between the phenyl ring on the phenylarsonic acid molecule and its closest symmetry equivalent.

We believe that the unexpected stoichiometry of the crystal is due to the balancing between hydrogen bond donors and acceptors: Hydrogen arsonate ions have two hydrogen bond acceptor sites and one hydrogen bond donor site, whereas arsonic acid molecules have two hydrogen bond donor sites and one hydrogen bond acceptor site, so that all sites with a potential for hydrogen bonding are saturated at a 1:1 stoichiometry between neutral acid and monodeprotonated anion. The resulting assembly is the least hydrophilic and therefore the first to crystallize from a concentrated aqueous solution containing a bulky hydrophobic cation.

### Experimental

Phenylarsonic acid (10 mmol, 2.02 g) was dissolved in 1 M aqueous tetrabutylammonium hydroxide solution (10 mmol, 10 ml). The colourless oil obtained by evaporation of the solution *in vacuo* formed large colourless crystals on standing at room temperature for 7 days.

### Refinement

H atoms were located in Fourier difference maps and their positions and displacement parameters were refined independently. Modelled C–H bond lengths vary from 0.81 (3) Å to 1.01 (2) Å due to libration effects. Modelled O–H bond lengths vary from 0.75 (3) Å to 0.96 (4) Å due to strong hydrogen bonding. The variations in As–O bond length are consistent with the resulting protonation of the arsonic acid molecules. Several reflections were omitted from the final refinement owing to poor agreement.

### Crystal data

**Table:**

C_16_H_36_N^+^·C_6_H_6_AsO_3_^−^·C_6_H_7_AsO_3_	*Z* = 2
*M**_r_* = 645.52	*F*(000) = 676
Triclinic, *P*1	*D*_x_ = 1.393 Mg m^−^^3^
Hall symbol: -P 1	Mo *K*α radiation, λ = 0.71073 Å
*a* = 9.035 (2) Å	Cell parameters from 232 reflections
*b* = 10.137 (3) Å	θ = 2.3–27.2°
*c* = 18.789 (5) Å	µ = 2.21 mm^−^^1^
α = 94.005 (5)°	*T* = 120 K
β = 97.749 (4)°	Block, colourless
γ = 114.289 (4)°	0.5 × 0.3 × 0.1 mm
*V* = 1539.2 (7) Å^3^	

### Data collection

**Table:**

Bruker SMART APEX diffractometer	7513 independent reflections
Radiation source: fine-focus sealed tube	6769 reflections with *I* > 2σ(*I*)
Graphite monochromator	*R*_int_ = 0.023
φ and ω scans	θ_max_ = 28.3°, θ_min_ = 2.2°
Absorption correction: multi-scan (*SADABS*; Bruker, 1997)	*h* = −12→12
*T*_min_ = 0.291, *T*_max_ = 0.809	*k* = −13→13
15253 measured reflections	*l* = −23→24

### Refinement

**Table:**

Refinement on *F*^2^	Primary atom site location: structure-invariant direct methods
Least-squares matrix: full	Secondary atom site location: difference Fourier map
*R*[*F*^2^ > 2σ(*F*^2^)] = 0.026	Hydrogen site location: difference Fourier map
*wR*(*F*^2^) = 0.070	All H-atom parameters refined
*S* = 1.03	*w* = 1/[σ^2^(*F*_o_^2^) + (0.0421*P*)^2^ + 0.2175*P*] where *P* = (*F*_o_^2^ + 2*F*_c_^2^)/3
7513 reflections	(Δ/σ)_max_ = 0.001
530 parameters	Δρ_max_ = 0.61 e Å^−^^3^
0 restraints	Δρ_min_ = −0.71 e Å^−^^3^

### Special details

**Table:**

Geometry. All e.s.d.'s (except the e.s.d. in the dihedral angle between two l.s. planes) are estimated using the full covariance matrix. The cell e.s.d.'s are taken into account individually in the estimation of e.s.d.'s in distances, angles and torsion angles; correlations between e.s.d.'s in cell parameters are only used when they are defined by crystal symmetry. An approximate (isotropic) treatment of cell e.s.d.'s is used for estimating e.s.d.'s involving l.s. planes.
Refinement. Refinement of *F*^2^ against ALL reflections. The weighted *R*-factor *wR* and goodness of fit *S* are based on *F*^2^, conventional *R*-factors *R* are based on *F*, with *F* set to zero for negative *F*^2^. The threshold expression of *F*^2^ > σ(*F*^2^) is used only for calculating *R*-factors(gt) *etc*. and is not relevant to the choice of reflections for refinement. *R*-factors based on *F*^2^ are statistically about twice as large as those based on *F*, and *R*- factors based on ALL data will be even larger.

### Fractional atomic coordinates and isotropic or equivalent isotropic displacement parameters (Å^2^)

**Table:**

	*x*	*y*	*z*	*U*_iso_*/*U*_eq_	
As1	0.983720 (17)	0.434497 (16)	0.191058 (8)	0.01665 (5)	
O11	0.84503 (14)	0.41025 (14)	0.24937 (6)	0.0253 (2)	
H11	0.799 (3)	0.458 (3)	0.2399 (15)	0.060 (8)*	
O12	1.13065 (13)	0.60598 (11)	0.20887 (6)	0.0208 (2)	
O13	1.05793 (13)	0.31108 (12)	0.20416 (6)	0.0218 (2)	
N1	1.04658 (14)	0.94499 (13)	0.26111 (7)	0.0157 (2)	
C11	0.86611 (17)	0.39575 (16)	0.09453 (8)	0.0193 (3)	
C12	0.9186 (2)	0.33147 (18)	0.04140 (9)	0.0248 (3)	
H12	1.011 (3)	0.312 (2)	0.0525 (12)	0.036 (5)*	
C13	0.8426 (2)	0.3079 (2)	−0.02994 (10)	0.0301 (4)	
H13	0.880 (2)	0.268 (2)	−0.0679 (11)	0.030 (5)*	
C14	0.7155 (2)	0.3475 (2)	−0.04864 (10)	0.0322 (4)	
H14	0.674 (3)	0.334 (2)	−0.0930 (12)	0.034 (6)*	
C15	0.6630 (2)	0.4103 (2)	0.00444 (11)	0.0379 (4)	
H15	0.571 (3)	0.437 (2)	−0.0117 (12)	0.044 (6)*	
C16	0.7377 (2)	0.4351 (2)	0.07646 (10)	0.0292 (4)	
H16	0.707 (3)	0.473 (2)	0.1076 (12)	0.034 (6)*	
C31	1.06254 (18)	0.83454 (16)	0.30861 (8)	0.0171 (3)	
H31A	0.950 (2)	0.764 (2)	0.3068 (10)	0.018 (4)*	
H31B	1.115 (2)	0.790 (2)	0.2842 (10)	0.019 (4)*	
C32	1.1517 (2)	0.89580 (18)	0.38583 (9)	0.0222 (3)	
H32A	1.089 (2)	0.926 (2)	0.4123 (11)	0.026 (5)*	
H32B	1.253 (2)	0.982 (2)	0.3874 (10)	0.021 (4)*	
C33	1.1868 (2)	0.7791 (2)	0.42250 (10)	0.0281 (3)	
H33A	1.261 (3)	0.751 (2)	0.3952 (12)	0.035 (5)*	
H33B	1.241 (3)	0.822 (2)	0.4668 (12)	0.034 (5)*	
C34	1.0341 (3)	0.6426 (2)	0.42641 (12)	0.0406 (5)	
H34A	1.060 (3)	0.580 (3)	0.4569 (13)	0.045 (6)*	
H34B	0.988 (3)	0.590 (3)	0.3788 (15)	0.050 (7)*	
H34C	0.962 (3)	0.675 (2)	0.4455 (12)	0.038 (6)*	
C41	0.93601 (17)	0.85567 (16)	0.19096 (8)	0.0167 (3)	
H41A	0.835 (2)	0.799 (2)	0.2031 (9)	0.015 (4)*	
H41B	0.984 (2)	0.7952 (19)	0.1749 (9)	0.014 (4)*	
C42	0.90926 (19)	0.94151 (17)	0.13157 (8)	0.0199 (3)	
H42A	0.870 (2)	1.012 (2)	0.1467 (10)	0.023 (5)*	
H42B	1.014 (3)	1.004 (2)	0.1162 (12)	0.037 (6)*	
C43	0.7900 (2)	0.8353 (2)	0.06719 (9)	0.0288 (3)	
H43A	0.828 (3)	0.767 (3)	0.0487 (12)	0.044 (6)*	
H43B	0.692 (3)	0.781 (3)	0.0840 (12)	0.039 (6)*	
C44	0.7506 (2)	0.9096 (2)	0.00493 (10)	0.0334 (4)	
H44A	0.677 (3)	0.839 (3)	−0.0324 (15)	0.054 (7)*	
H44B	0.847 (3)	0.971 (3)	−0.0122 (13)	0.050 (7)*	
H44C	0.698 (3)	0.973 (3)	0.0199 (13)	0.047 (6)*	
C51	1.21333 (17)	1.05089 (16)	0.24719 (8)	0.0180 (3)	
H51A	1.280 (2)	1.095 (2)	0.2939 (10)	0.018 (4)*	
H51B	1.192 (2)	1.121 (2)	0.2232 (10)	0.016 (4)*	
C52	1.30155 (18)	0.98468 (17)	0.20316 (9)	0.0209 (3)	
H52A	1.237 (2)	0.947 (2)	0.1528 (11)	0.026 (5)*	
H52B	1.310 (2)	0.904 (2)	0.2218 (11)	0.024 (5)*	
C53	1.47142 (19)	1.10091 (18)	0.19840 (10)	0.0224 (3)	
H53A	1.462 (2)	1.176 (2)	0.1776 (11)	0.027 (5)*	
H53B	1.530 (2)	1.133 (2)	0.2446 (11)	0.022 (5)*	
C54	1.5617 (2)	1.0398 (2)	0.15297 (11)	0.0288 (4)	
H54A	1.664 (3)	1.110 (2)	0.1490 (12)	0.035 (5)*	
H54B	1.506 (3)	1.010 (2)	0.1048 (13)	0.036 (6)*	
H54C	1.567 (3)	0.956 (3)	0.1718 (13)	0.044 (6)*	
C61	0.97536 (18)	1.03791 (16)	0.29762 (8)	0.0182 (3)	
H61A	0.972 (2)	1.107 (2)	0.2659 (10)	0.017 (4)*	
H61B	1.062 (2)	1.094 (2)	0.3409 (10)	0.018 (4)*	
C62	0.81020 (19)	0.95365 (18)	0.31980 (9)	0.0213 (3)	
H62A	0.810 (2)	0.882 (2)	0.3471 (11)	0.029 (5)*	
H62B	0.725 (2)	0.900 (2)	0.2774 (11)	0.028 (5)*	
C63	0.7615 (2)	1.0596 (2)	0.36162 (10)	0.0274 (3)	
H63A	0.845 (3)	1.116 (2)	0.4042 (12)	0.037 (6)*	
H63B	0.765 (3)	1.141 (3)	0.3339 (13)	0.041 (6)*	
C64	0.5945 (2)	0.9811 (2)	0.38350 (11)	0.0338 (4)	
H64A	0.559 (3)	1.040 (3)	0.4060 (13)	0.044 (6)*	
H64B	0.599 (3)	0.917 (3)	0.4161 (16)	0.070 (9)*	
H64C	0.512 (3)	0.928 (3)	0.3427 (13)	0.043 (6)*	
As2	0.478325 (17)	0.517474 (16)	0.248301 (8)	0.01779 (5)	
O21	0.34772 (15)	0.38567 (13)	0.17947 (6)	0.0231 (2)	
H21	0.260 (4)	0.365 (3)	0.1849 (15)	0.062 (9)*	
O22	0.42750 (14)	0.66150 (12)	0.24787 (7)	0.0251 (2)	
H22	0.317 (5)	0.641 (4)	0.236 (2)	0.112 (13)*	
O23	0.67109 (13)	0.56498 (13)	0.23897 (7)	0.0285 (3)	
C21	0.43938 (19)	0.44095 (16)	0.33777 (8)	0.0211 (3)	
C22	0.3014 (2)	0.4323 (2)	0.36604 (9)	0.0287 (3)	
H22A	0.232 (3)	0.472 (2)	0.3414 (12)	0.037 (6)*	
C23	0.2713 (3)	0.3712 (2)	0.42920 (11)	0.0416 (5)	
H23	0.196 (4)	0.366 (4)	0.4490 (18)	0.081 (11)*	
C24	0.3770 (3)	0.3205 (2)	0.46396 (11)	0.0476 (6)	
H24	0.362 (3)	0.277 (3)	0.5068 (16)	0.066 (8)*	
C25	0.5133 (3)	0.3297 (2)	0.43635 (11)	0.0456 (5)	
H25	0.585 (3)	0.299 (3)	0.4570 (15)	0.059 (8)*	
C26	0.5467 (2)	0.38999 (19)	0.37277 (10)	0.0312 (4)	
H26	0.638 (3)	0.400 (2)	0.3565 (11)	0.031 (5)*	

### Atomic displacement parameters (Å^2^)

**Table:**

	*U*^11^	*U*^22^	*U*^33^	*U*^12^	*U*^13^	*U*^23^
As1	0.01668 (8)	0.01561 (8)	0.01751 (8)	0.00714 (6)	0.00178 (6)	0.00218 (6)
O11	0.0238 (5)	0.0326 (7)	0.0222 (6)	0.0133 (5)	0.0079 (4)	0.0065 (5)
O12	0.0195 (5)	0.0149 (5)	0.0273 (6)	0.0079 (4)	0.0016 (4)	−0.0001 (4)
O13	0.0214 (5)	0.0158 (5)	0.0293 (6)	0.0094 (4)	0.0017 (4)	0.0055 (4)
N1	0.0160 (5)	0.0137 (6)	0.0172 (6)	0.0061 (5)	0.0031 (4)	0.0024 (5)
C11	0.0193 (6)	0.0153 (7)	0.0198 (7)	0.0046 (6)	0.0014 (5)	0.0028 (6)
C12	0.0269 (7)	0.0247 (8)	0.0227 (8)	0.0115 (7)	0.0032 (6)	0.0018 (6)
C13	0.0365 (9)	0.0262 (9)	0.0222 (8)	0.0086 (7)	0.0055 (7)	−0.0014 (7)
C14	0.0342 (9)	0.0303 (9)	0.0210 (8)	0.0062 (7)	−0.0058 (7)	0.0014 (7)
C15	0.0343 (9)	0.0462 (11)	0.0337 (10)	0.0229 (9)	−0.0086 (8)	0.0001 (9)
C16	0.0276 (8)	0.0351 (9)	0.0263 (9)	0.0178 (7)	−0.0015 (7)	−0.0032 (7)
C31	0.0195 (6)	0.0144 (7)	0.0184 (7)	0.0080 (6)	0.0032 (5)	0.0036 (5)
C32	0.0258 (7)	0.0216 (8)	0.0187 (7)	0.0109 (7)	0.0002 (6)	0.0016 (6)
C33	0.0337 (9)	0.0297 (9)	0.0212 (8)	0.0151 (7)	−0.0009 (7)	0.0068 (7)
C34	0.0480 (11)	0.0385 (11)	0.0358 (11)	0.0149 (10)	0.0126 (9)	0.0215 (9)
C41	0.0168 (6)	0.0161 (7)	0.0157 (7)	0.0059 (6)	0.0019 (5)	0.0016 (5)
C42	0.0205 (7)	0.0211 (7)	0.0185 (7)	0.0089 (6)	0.0041 (6)	0.0045 (6)
C43	0.0336 (9)	0.0290 (9)	0.0204 (8)	0.0125 (8)	−0.0028 (7)	0.0023 (7)
C44	0.0339 (9)	0.0449 (11)	0.0219 (8)	0.0178 (9)	0.0008 (7)	0.0086 (8)
C51	0.0171 (6)	0.0135 (7)	0.0211 (7)	0.0043 (5)	0.0036 (5)	0.0025 (6)
C52	0.0171 (6)	0.0183 (7)	0.0263 (8)	0.0066 (6)	0.0049 (6)	0.0009 (6)
C53	0.0186 (7)	0.0187 (7)	0.0289 (9)	0.0066 (6)	0.0054 (6)	0.0038 (7)
C54	0.0220 (7)	0.0248 (8)	0.0429 (11)	0.0109 (7)	0.0125 (7)	0.0068 (8)
C61	0.0202 (6)	0.0162 (7)	0.0201 (7)	0.0093 (6)	0.0044 (6)	0.0019 (6)
C62	0.0210 (7)	0.0210 (7)	0.0246 (8)	0.0107 (6)	0.0065 (6)	0.0035 (6)
C63	0.0237 (7)	0.0305 (9)	0.0292 (9)	0.0140 (7)	0.0049 (7)	−0.0040 (7)
C64	0.0258 (8)	0.0432 (11)	0.0339 (10)	0.0170 (8)	0.0078 (7)	−0.0044 (9)
As2	0.01481 (8)	0.01589 (8)	0.02339 (9)	0.00662 (6)	0.00487 (6)	0.00389 (6)
O21	0.0246 (6)	0.0215 (6)	0.0224 (6)	0.0101 (5)	0.0022 (5)	0.0002 (4)
O22	0.0236 (5)	0.0161 (5)	0.0366 (7)	0.0103 (5)	0.0028 (5)	0.0032 (5)
O23	0.0171 (5)	0.0243 (6)	0.0462 (7)	0.0081 (5)	0.0123 (5)	0.0083 (5)
C21	0.0233 (7)	0.0153 (7)	0.0203 (7)	0.0056 (6)	−0.0006 (6)	0.0005 (6)
C22	0.0272 (8)	0.0300 (9)	0.0212 (8)	0.0054 (7)	0.0033 (6)	−0.0007 (7)
C23	0.0475 (11)	0.0354 (11)	0.0253 (9)	0.0002 (9)	0.0123 (9)	−0.0008 (8)
C24	0.0809 (16)	0.0269 (10)	0.0188 (9)	0.0090 (10)	0.0026 (10)	0.0033 (7)
C25	0.0738 (15)	0.0286 (10)	0.0294 (10)	0.0248 (11)	−0.0150 (10)	−0.0004 (8)
C26	0.0371 (9)	0.0228 (8)	0.0309 (9)	0.0147 (7)	−0.0062 (7)	−0.0015 (7)

### Geometric parameters (Å, º)

**Table:**

As1—O13	1.6625 (10)	C44—H44C	0.99 (2)
As1—O12	1.6723 (11)	C51—C52	1.520 (2)
As1—O11	1.7279 (11)	C51—H51A	0.956 (19)
As1—C11	1.9001 (16)	C51—H51B	0.941 (18)
O11—H11	0.77 (3)	C52—C53	1.522 (2)
N1—C41	1.5130 (18)	C52—H52A	1.00 (2)
N1—C61	1.5163 (17)	C52—H52B	0.94 (2)
N1—C51	1.5185 (18)	C53—C54	1.519 (2)
N1—C31	1.5194 (18)	C53—H53A	0.91 (2)
C11—C16	1.383 (2)	C53—H53B	0.91 (2)
C11—C12	1.392 (2)	C54—H54A	0.92 (2)
C12—C13	1.378 (2)	C54—H54B	0.94 (2)
C12—H12	0.94 (2)	C54—H54C	0.96 (2)
C13—C14	1.376 (3)	C61—C62	1.516 (2)
C13—H13	0.96 (2)	C61—H61A	0.954 (18)
C14—C15	1.383 (3)	C61—H61B	1.000 (18)
C14—H14	0.84 (2)	C62—C63	1.526 (2)
C15—C16	1.384 (3)	C62—H62A	0.92 (2)
C15—H15	1.00 (2)	C62—H62B	0.97 (2)
C16—H16	0.82 (2)	C63—C64	1.518 (2)
C31—C32	1.512 (2)	C63—H63A	0.98 (2)
C31—H31A	0.966 (19)	C63—H63B	1.00 (2)
C31—H31B	0.922 (18)	C64—H64A	0.89 (2)
C32—C33	1.529 (2)	C64—H64B	0.93 (3)
C32—H32A	0.929 (19)	C64—H64C	0.94 (2)
C32—H32B	0.96 (2)	As2—O23	1.6432 (11)
C33—C34	1.515 (3)	As2—O22	1.7013 (11)
C33—H33A	1.01 (2)	As2—O21	1.7030 (12)
C33—H33B	0.89 (2)	As2—C21	1.9153 (16)
C34—H34A	0.96 (3)	O21—H21	0.75 (3)
C34—H34B	0.95 (3)	O22—H22	0.93 (4)
C34—H34C	0.94 (2)	C21—C26	1.388 (2)
C41—C42	1.515 (2)	C21—C22	1.393 (2)
C41—H41A	0.930 (18)	C22—C23	1.385 (3)
C41—H41B	0.943 (17)	C22—H22A	0.96 (2)
C42—C43	1.515 (2)	C23—C24	1.372 (4)
C42—H42A	0.964 (19)	C23—H23	0.81 (3)
C42—H42B	1.00 (2)	C24—C25	1.371 (4)
C43—C44	1.515 (2)	C24—H24	0.94 (3)
C43—H43A	0.95 (2)	C25—C26	1.392 (3)
C43—H43B	0.94 (2)	C25—H25	0.88 (3)
C44—H44A	0.93 (3)	C26—H26	0.89 (2)
C44—H44B	0.95 (3)		
			
O13—As1—O12	112.71 (6)	H44A—C44—H44C	108 (2)
O13—As1—O11	106.12 (6)	H44B—C44—H44C	106 (2)
O12—As1—O11	109.42 (6)	N1—C51—C52	115.86 (12)
O13—As1—C11	109.58 (6)	N1—C51—H51A	106.0 (11)
O12—As1—C11	110.72 (6)	C52—C51—H51A	109.5 (10)
O11—As1—C11	108.09 (6)	N1—C51—H51B	104.9 (11)
As1—O11—H11	108 (2)	C52—C51—H51B	110.5 (10)
C41—N1—C61	111.98 (11)	H51A—C51—H51B	109.9 (15)
C41—N1—C51	110.79 (11)	C51—C52—C53	109.94 (12)
C61—N1—C51	105.97 (11)	C51—C52—H52A	109.8 (10)
C41—N1—C31	105.49 (11)	C53—C52—H52A	108.1 (11)
C61—N1—C31	111.07 (11)	C51—C52—H52B	112.6 (12)
C51—N1—C31	111.65 (11)	C53—C52—H52B	110.7 (12)
C16—C11—C12	120.67 (15)	H52A—C52—H52B	105.6 (16)
C16—C11—As1	121.87 (12)	C54—C53—C52	111.50 (14)
C12—C11—As1	117.40 (11)	C54—C53—H53A	107.9 (12)
C13—C12—C11	119.57 (15)	C52—C53—H53A	110.2 (12)
C13—C12—H12	119.0 (13)	C54—C53—H53B	109.1 (11)
C11—C12—H12	121.2 (13)	C52—C53—H53B	107.3 (12)
C14—C13—C12	120.28 (16)	H53A—C53—H53B	110.8 (17)
C14—C13—H13	118.4 (12)	C53—C54—H54A	112.0 (13)
C12—C13—H13	121.2 (12)	C53—C54—H54B	111.1 (13)
C13—C14—C15	119.90 (17)	H54A—C54—H54B	103.3 (19)
C13—C14—H14	117.8 (14)	C53—C54—H54C	109.8 (14)
C15—C14—H14	122.3 (14)	H54A—C54—H54C	112.9 (19)
C14—C15—C16	120.79 (17)	H54B—C54—H54C	107.5 (19)
C14—C15—H15	117.1 (13)	C62—C61—N1	115.27 (12)
C16—C15—H15	122.1 (13)	C62—C61—H61A	112.6 (10)
C11—C16—C15	118.79 (16)	N1—C61—H61A	106.8 (10)
C11—C16—H16	120.8 (15)	C62—C61—H61B	111.0 (10)
C15—C16—H16	120.4 (15)	N1—C61—H61B	103.2 (10)
C32—C31—N1	115.79 (12)	H61A—C61—H61B	107.2 (15)
C32—C31—H31A	111.6 (11)	C61—C62—C63	109.42 (13)
N1—C31—H31A	104.5 (11)	C61—C62—H62A	112.9 (12)
C32—C31—H31B	111.6 (11)	C63—C62—H62A	110.1 (12)
N1—C31—H31B	104.9 (11)	C61—C62—H62B	110.8 (12)
H31A—C31—H31B	107.8 (15)	C63—C62—H62B	109.8 (11)
C31—C32—C33	109.85 (13)	H62A—C62—H62B	103.7 (17)
C31—C32—H32A	110.9 (12)	C64—C63—C62	111.56 (15)
C33—C32—H32A	108.9 (12)	C64—C63—H63A	110.2 (13)
C31—C32—H32B	111.2 (11)	C62—C63—H63A	112.0 (12)
C33—C32—H32B	110.5 (11)	C64—C63—H63B	111.4 (13)
H32A—C32—H32B	105.5 (16)	C62—C63—H63B	111.7 (13)
C34—C33—C32	114.41 (15)	H63A—C63—H63B	99.4 (18)
C34—C33—H33A	109.1 (12)	C63—C64—H64A	114.5 (16)
C32—C33—H33A	108.3 (12)	C63—C64—H64B	110.2 (17)
C34—C33—H33B	110.2 (14)	H64A—C64—H64B	105 (2)
C32—C33—H33B	105.8 (14)	C63—C64—H64C	111.0 (14)
H33A—C33—H33B	108.9 (18)	H64A—C64—H64C	106 (2)
C33—C34—H34A	111.7 (14)	H64B—C64—H64C	109 (2)
C33—C34—H34B	108.6 (15)	O23—As2—O22	111.86 (6)
H34A—C34—H34B	108 (2)	O23—As2—O21	110.46 (6)
C33—C34—H34C	105.8 (14)	O22—As2—O21	107.93 (6)
H34A—C34—H34C	110 (2)	O23—As2—C21	110.94 (7)
H34B—C34—H34C	112 (2)	O22—As2—C21	107.65 (6)
N1—C41—C42	116.09 (12)	O21—As2—C21	107.84 (6)
N1—C41—H41A	106.0 (11)	As2—O21—H21	109 (2)
C42—C41—H41A	108.8 (11)	As2—O22—H22	118 (2)
N1—C41—H41B	105.8 (10)	C26—C21—C22	120.47 (17)
C42—C41—H41B	110.2 (10)	C26—C21—As2	119.45 (13)
H41A—C41—H41B	109.8 (15)	C22—C21—As2	120.05 (12)
C43—C42—C41	108.80 (13)	C23—C22—C21	119.34 (18)
C43—C42—H42A	110.4 (11)	C23—C22—H22A	122.5 (13)
C41—C42—H42A	113.6 (11)	C21—C22—H22A	118.1 (13)
C43—C42—H42B	109.7 (13)	C24—C23—C22	120.4 (2)
C41—C42—H42B	112.0 (13)	C24—C23—H23	116 (2)
H42A—C42—H42B	102.2 (17)	C22—C23—H23	124 (2)
C44—C43—C42	113.39 (16)	C25—C24—C23	120.3 (2)
C44—C43—H43A	107.8 (14)	C25—C24—H24	115.5 (17)
C42—C43—H43A	112.9 (14)	C23—C24—H24	124.2 (17)
C44—C43—H43B	108.8 (14)	C24—C25—C26	120.7 (2)
C42—C43—H43B	106.7 (14)	C24—C25—H25	123.4 (18)
H43A—C43—H43B	106.9 (19)	C26—C25—H25	115.9 (18)
C43—C44—H44A	109.2 (16)	C21—C26—C25	118.77 (19)
C43—C44—H44B	112.1 (15)	C21—C26—H26	120.8 (14)
H44A—C44—H44B	110 (2)	C25—C26—H26	120.3 (14)
C43—C44—H44C	111.2 (14)		

### Hydrogen-bond geometry (Å, º)

**Table:**

*D*—H···*A*	*D*—H	H···*A*	*D*···*A*	*D*—H···*A*
O11—H11···O23	0.77 (3)	1.88 (3)	2.6375 (17)	166 (3)
O21—H21···O13^i^	0.75 (3)	1.78 (3)	2.5280 (17)	176 (3)
O22—H22···O12^i^	0.93 (4)	1.57 (4)	2.4936 (17)	176 (4)

Symmetry code: (i) *x*−1, *y*, *z*.
